# The unexpected PD-L1 suppression function of celery-derived extracellular vesicles improves lung cancer chemotherapy efficacy

**DOI:** 10.20517/evcna.2023.75

**Published:** 2024-11-09

**Authors:** Xin Lu, Ziwen Yu, Junjun Wang, Ao Tian, Tingyu Wu, Yirui Cheng, Qing Han, Fan Li, Weiliang Xia

**Affiliations:** State Key Laboratory of Systems Medicine for Cancer, Renji Hospital, School of Medicine and School of Biomedical Engineering, Shanghai Jiao Tong University, Shanghai 200030, China.; ^#^Authors contributed equally.

**Keywords:** Celery-derived extracellular vesicles (CDEVs), PD-L1, CD8+ T cell, paclitaxel (PTX)

## Abstract

**Aim:** The article explores celery-derived extracellular vesicles (CDEVs), characterized by high cellular uptake, low immunogenicity, and high stability, as a therapeutic strategy for antitumor nanomedicines.

**Methods:** The methods employed in this study include *in vitro* cell experiments such as co-culture, Western Blot, and flow cytometry. *In vivo* experiments were conducted in C57BL/6 tumor-bearing mice subcutaneously injected with Lewis lung carcinoma (LLC) cells. The experiments encompassed parameters such as survival rate, body weight, tumor size, flow cytometry, immunohistochemistry, and spectral live imaging system.

**Results:** Our study revealed that CDEVs could be used as drugs to effectively downregulate the phosphorylated signal transducer and activator of transcription 3 (p-STAT3)/programmed cell death ligand 1 (PD-L1) axis in lung cancer cells. In co-culture experiments, CDEVs were observed to impede the expression of PD-L1, thereby interfering with the interaction between PD-L1 and programmed death 1 (PD-1) and subsequently preventing the suppression of T cells. In *in vivo* distribution experiments, CDEVs loaded with paclitaxel (PTX) demonstrated better tumor targeting capabilities. Remarkably, following CDEVs-PTX treatment, CD8+ T cell levels in mice were increased, presumably leading to improved antitumor effects.

**Conclusion:** CDEVs not only serve as drug carriers but also function as drugs themselves; as such, through a single administration of CDEVs, it is possible to combine immunotherapy and chemotherapy to achieve better effects between the two, providing a more comprehensive and effective cancer treatment strategy that promises to improve treatment outcomes and reduce the adverse effects of therapy.

## INTRODUCTION

Lung cancer, which predominantly includes non-small-cell lung cancer (NSCLC) and small-cell lung cancer (SCLC), remains the leading cause of cancer-related deaths despite advances in treatments like surgery, radiotherapy, chemotherapy, and targeted therapy^[1-3]^. The effectiveness of immunotherapy, particularly programmed death 1 (PD-1)/programmed cell death-Ligand 1 (PD-L1) immune checkpoint inhibitors, which has become a key treatment option, depends on the tumor immune microenvironment, such as the infiltration of CD8+ T cells into the tumor^[4,5]^. These T cells are often suppressed by PD-L1 expressed on tumor cells, promoting immune evasion^[6]^. Additionally, the activity of the Pdcd-1L1 promoter (encoding PD-L1) can be enhanced by activated signal transducer and activator of transcription 3 (STAT3), thereby promoting the expression of PD-L1 protein^[7]^. Based on this, targeting the p-STAT3/PD-L1 pathway in lung cancer treatment may benefit cancer patients by enhancing antitumor immunity. However, challenges such as immune tolerance and T cell depletion can undermine the effectiveness of monotherapy, underscoring the need for combined treatment approaches in lung cancer therapy^[8]^.

Combining immunotherapy-based treatments requires addressing two key aspects: expanding the initial T cell population or reducing the tumor burden^[9]^. Clinical research into the use of chemotherapy in combination with immunotherapy has been conducted in the context of lung cancer. In two phase II clinical trials, a phased approach involving the combination of pembrolizumab with paclitaxel (PTX) and cisplatin demonstrated improved efficacy^[10-12]^. This highlights the significance of employing a combination of chemotherapy and immunotherapy in the treatment of lung cancer. On the other hand, chemotherapy for lung cancer is effective but causes significant side effects and has issues like poor solubility and bioavailability^[13,14]^. Encapsulating these drugs in nanoparticles such as extracellular vesicles^[15]^ and liposomes^[16]^ can enhance tumor targeting and reduce side effects. However, the high production costs and risks associated with synthetic nanoparticles are prompting research into Plant-derived extracellular vesicles (PDEVs) as an alternative^[17]^.

PDEVs are nanosized vesicles that are rich in lipids, proteins, RNA, and natural active ingredients^[18]^. The low cost, low immunogenicity, high stability, and high tissue penetrability of PDEVs make them more suitable as drug carriers compared to other nanoparticles^[19]^. Wang *et al*. utilized grapefruit-derived extracellular vesicles to transport chemotherapy drugs, siRNA, proteins, and other substances to various types of cells^[20]^. Moreover, PDEVs exhibit therapeutic effects as drug therapeutic agents due to their natural cargo content^[21]^. They have shown tremendous potential in the treatment of colitis, anticancer therapies, and prevention of alcohol-induced liver damage^[22]^.

Celery (Apium graveolens L) belongs to the *Apiaceae* family and is rich in vitamins, carotenoids, proteins, and other nutrients. It also serves as a good source of flavonoids, alkaloids, terpenoids, and phenolic acids^[23]^. Celery demonstrates a range of pharmacological activities, including antioxidant, anticancer, anti-inflammatory, and cardioprotective effects^[24]^. In our previous study, we found that PDEVs derived from celery-derived extracellular vesicles (CDEVs) outperformed liposomes as drug carriers, showing superior drug delivery capabilities^[25]^. In this study, we have discovered that CDEVs inhibit PD-L1 expression in lung cancer cells, presumably leading to reduced PD-1 and PD-L1 interactions, thus preventing immune evasion of tumor cells. In addition to serving as an immunomodulator, CDEVs also exhibit drug carrier capabilities. When loaded with PTX is encapsulated in CDEVs, it can accumulate more effectively at the tumor site, leading to prolonged survival in tumor-bearing mice and aiding in tumor inhibition.

## METHODS

### The isolation and extraction of CDEVs

Celery (A. graveolens L.) was sourced from a local vegetable market in China. The celery sample was placed in Shanghai Chenshan Herbarium(CSH) with the assistance of Dr. Xiaochen Li from CSH^[25]^. After washing, the celery was processed using a wall-breaking blender to extract the juice, yielding approximately 500 mL per kilogram. This juice was then subjected to a series of three centrifugations to yield the supernatant. The three rounds of centrifugation by centrifuge (Beckman, Avanti JXN-30) were conducted under the following conditions: 2,000 g for 20 min, 5,000 g for 30 min, and 10,000 g for 1 h. Following this, an ultracentrifuge (Beckman, Optima XPN) was employed at 100,000 g for 2 h, with the supernatant subsequently discarded. All centrifugation procedures were performed at 4 °C. The resulting pellet, referred to as the samples, was then resuspended in clean phosphate buffered saline (PBS). The samples were transferred into 30%, 45%, and 60% sucrose solutions at 100,000 g for 1 h using density gradient centrifugation (Excluding the time for centrifuge program ramp up and down), and the intermediate layers of 30% ~ 45% sucrose solutions were collected. The samples were washed with an appropriate amount of PBS and subsequently centrifuged at 100,000 g for 1 h. CDEVs were resuspended in PBS.

### Reagents and drugs

Polyacrylamide Gel Electrophoresis (PAGE) Gel Fast Preparation Kit, Cat#PG112, Epizyme; Formvar/carbon film coated grid, Cat#BZ110223a, Beijing Zhongjingkeyi technology; Multicolor Prestained Protein Ladder, Cat#WJ101, Beyotime Biotechnology; DiL, Cat#C1036, Beyotime Biotechnology; DiR, DiIC_18_(7), Cat#D12731, Invitrogen; PTX, Cat#HY-B0015, MedChemExpress; Cell Activation Cocktail (with Brefeldin A), Cat#423303, Biolegend; Red blood cell lysis buffer, Cat#C3702, Beyotime; Fluorescein Isothiocyanate (FITC) anti-mouse IL-2, Cat#503805, Biolegend; Murine interferon-γ (IFN-γ), Cat#575302, Biolegend; Zombie Aqua Fixable Viability, Cat#423101, Biolegend; Peridinin-Chlorophyll-Protein Complex-Cyanine 5.5 (PERCP-CY5.5) anti-human PD-1, Cat#329913, Biolegend; AF700 anti-human/mouse GranzymeB, Cat#372221, Biolegend; BV605 anti-mouse IFN-γ, Cat#505839, Biolegend; PERCP-CY5.5 anti-mouse CD8α, Cat#551162, BD Biosciences; FITC anti-mouse CD4, Cat#100405, Biolegend; PE-CY7 anti-mouse tumor necrosis factor (TNF-α), Cat#506323, Biolegend; Anti-Glyceraldehyde-3-Phosphate Dehydrogenase (GAPDH) antibody, Cat#ab59164, Abcam; Anti-p-STAT3 antibody, Cat#ab86430, Abcam; Anti-PD-L1 antibody, Cat#ab205921, Abcam; FITC-PTX, Cat#Q-0066348, Xi’an Qiyue Biology; PTX, Cat#HY-B0015, MedChemExpress; Phosphate Buffer Saline, Cytiva; Dulbecco’s Modified Eagle Medium (DMEM), Cytiva; RPMI, Cytiva; Penicillin-Streptomycin, Hyclone; Trypsin, Gibco; Fetal Bovine Serum (FBS), Lonsera; Uranyl acetate, Merck; Dimethyl Sulfoxide (DMSO), Thermo; Radio Immunoprecipitation Assay Buffer (RIPA), Cat#20-188, Millipore; Phenylmethylsulfonyl Fluoride (PMSF), Beyotime; BCA kit, Cat#23227, Thermo; Chemiluminescent imaging system, Cat#5200, Tanon; Bovine Serum Albumin (BSA), China Pharmaceutical; Loading buffer, Epizyme; Fixation buffer, Cat#B284704, Biolegend; Permeabilization buffer 10x, Cat#00-8333-56, Thermo; Anti-mouse IgG, Jackson; Anti-rabbit IgG, Jackson; Trichloromethane, China Pharmaceutical; Anti-fluorescence quenching sealer, Beyotime; Cleaved Caspase-3 (Asp175) Antibody, Cat#9661, Cell Signaling Technology (CST); Ki-67 (8D5) Mouse mAb, Cat#9449, CST.

### Nanoparticle tracking analysis

The highly concentrated CDEVs samples were diluted between 1,000- and 5,000-fold using PBS. The Nanoparticle Tracking Analysis (NTA) instrument (Particle Metrix, ZetaView) was calibrated using nanoparticle standards with a nominal diameter of 100 nm^[14]^. Following calibration, the NTA was utilized to measure the particle size and concentration of the CDEVs. The Zetaview NTA system from Particle Metrix was equipped with a high-sensitivity complementary metal-oxide-semiconductor (CMOS) camera, which had a resolution of 640 pixels × 480 pixels and an adjustable frame rate ranging from 1 to 60 Hz.

### Transmission electron microscope

CDEVs samples (10 μL) were deposited onto the surface of formvar/carbon film coated grid and allowed to stand for 3-5 min. Any excess liquid present on the surface was subsequently absorbed using air-laid paper. Following this, 1 μL uranyl acetate staining solution was added to the surface of formvar/carbon film coated grid and left undisturbed for a period ranging from 30 s to 1 min. After the removal of any excess stain on the surface, the sample was dried under an incandescent lamp. The morphology of the extracellular vesicles was then examined using transmission electron microscope (TEM) (Thermofisher, Tecnai G2 spirit Biotwin).

### Atomic force microscopy

CDEVs were diluted with ddH_2_O to a concentration of 1 × 10^10^ particles/mL and seeded onto a flat mica substrate, and then air-dried. Atomic force microscopy (AFM) (Bruker, FastScan Bio) was performed according to the manufacturer’s instructions. The Nanoscope and Dimension Stage controllers were powered on, the sharp nanoscale probe was installed, and the laser was aligned. After launching the Nanoscope software and setting up the experimental interface, the probe’s position was located, and scanning commenced. The probe moved across the sample, collecting data for image analysis. Data were processed using NanoScope Analysis software as follows: (1) Click “Flatten” in the 2D graphics interface, adjust to level 2, and modify the z threshold to highlight most particles; (2) Adjust image height and brightness by right-clicking the color bar, selecting “Color Scale”, and modifying the data scale to optimize brightness and contrast, then save the 2D image; (3) View and adjust the 3D image by left-clicking to rotate, then save; (4) Analyze CDEVs’ size, shape, and distribution from the 2D and 3D images.

### Immunohistochemistry

Paraffin sections were dewaxed in xylene (10 min, twice), then sequentially washed in anhydrous ethanol (twice), 95%, 85%, and 75% ethanol for 5 min each. After rinsing with water, sections were treated with 3% hydrogen peroxide for 10 min, followed by another rinse. PBS washes (twice) were performed before drying the tissue edges, which were circled with an immunohistochemical pen. A 10% goat serum was applied for blocking at 37 °C.

The primary antibody (Ki-67 and Cleaved Caspase-3), diluted as per instructions, was applied after 1 h of blocking. Samples were incubated overnight at 4 °C, then washed with PBS (3 times, 5 min each). The secondary antibody was applied for 1 h, followed by PBS washes (3 times, 5 min each). Specific affinity stable binding (SASB) (100-fold dilution) was added for 1 h. A DAB (3,3'-Diaminobenzidine)-based color developer was prepared with PBS, H_2_O_2_, and phosphate buffer, applied after drying the PBS around the tissue. Hematoxylin counterstaining was done at 37 °C for 5 min. After dehydration, slices were sealed with neutral resin.

Microscopic examination was performed, and ImageJ software was used for analysis. Automated scoring was done with Immunohistochemistry (IHC) Profiler, and positive and negative cells were counted using Trainable Weka Segmentation. Positive cell counts were calculated using the following formula:

The Percentage of positive cells (%) = (Number of positive cells/Number of positive plus negative cells) × 100%.

### Western blot

Cells harvested from six-well plates were lysed by adding RIPA buffer and scraping the adherent cells with a cell scraper. After incubating with agitation at 4 °C for 20 min, the samples were centrifuged at 12,000 rpm for 20 min. The supernatant, which represents the soluble protein sample, was then collected. Protein concentrations were subsequently measured using the BCA assay, and the volume of the sample was determined. The 10% sodium dodecyl sulfate polyacrylamide gel electrophoresis (SDS-PAGE) gels were prepared in advance. For each sample, 30 μg of protein was mixed with 5 μL of Loading Buffer and then denatured at 95 °C for 5 min. Depending on the sample volume, the thickness of the SDS-PAGE gel was chosen accordingly.

The treated protein samples were slowly loaded into the wells of the gel, followed by the addition of the marker. The gel was then subjected to electrophoresis at 120 V for 85 min. After electrophoresis, proteins were transferred onto nitrocellulose (NC) membranes using a wet transfer system. The transfer was performed at a constant current of 300 mA for approximately 80 min. Upon completion of the transfer, the membranes were blocked with 5% BSA for 1 h. Following a wash with Tris-buffered saline with Tween 20 (TBST), primary antibodies were applied, and the membranes were left to incubate overnight at 4 °C with gentle agitation. The primary antibodies for p-STAT3 and PD-L1 were diluted at a ratio of 1:1,000, while GAPDH was diluted at a ratio of 1:10,000. The next day, after removing the primary antibodies through washing, the membranes were treated with secondary antibodies diluted 1,000-fold. After 1 h, the secondary antibodies were washed off with TBST. Western blots were visualized using a chemiluminescent imaging system. The intensity of the protein bands was quantified and assessed using ImageJ software.

### Co-culture experiments

On the first day, Jurkat cells were grown in a medium without serum for 24 h. On the second day, Phorbol 12-Myristate 13-Acetate (PMA, 20 nM) was introduced to activate the Jurkat cells. For tumor cells, they were initially cultured in a 96-well plate on the first day. On the second day, CDEVs (1 × 10^11^ /well) /PBS and IFN-γ (100 ng/ml) were added to tumor cells. On the third day, any excess liquid in the 96-well plate was removed, followed by a wash with PBS (care was taken not to dislodge the cells). Subsequently, activated Jurkat cells were added (1.2 × 10^6^ /mL, 100 μL) to the 96-well plate with adherent tumor cells (HCC15 and H520). After a 48-h incubation period, Jurkat cells from the supernatant and adherent tumor cells were harvested for flow cytometry analysis.

### Flow cytometry

The cells or tissues were digested into single-cell suspensions and placed into 96-well U-bottom plates. For the digestion of adherent cells, trypsin was used. For the digestion of spleen tissue samples, mouse spleens were placed in DMEM medium without serum and antibiotics, and ground repeatedly with a pestle until no bright red tissue was visible. All the liquid was transferred to a 15 mL centrifuge tube with 14 mL of red blood cell lysis buffer, centrifuged at 400 g for 5 min in a pre-cooled centrifuge, and the supernatant was discarded. The cells were resuspended in 1.5 mL of PBS, filtered through a 70 μm filter, mixed, counted, and diluted to a concentration of 1 × 10^7^ /mL. 100 μL of the cell suspension was added to the 96-well U-bottom plate.

For the digestion of tumor tissue samples, tumor tissues were placed in a six-well plate and cut into approximately 2 mm pieces with scissors. Then, 2 mL of DMEM medium without serum and antibiotics was added, the samples were dispersed and mixed, and collagenase and hyaluronidase were added. The mixture was thoroughly stirred and incubated at 37 °C for 30 min. After digestion, complete medium was added to halt the reaction. The six-well plate was placed on ice, thoroughly ground, and filtered through a 70 μm filter. The cell suspension was mixed, counted, and diluted to a concentration of 1 × 10^7^ /mL. Subsequently, 100 μL of the cell suspension was added to the 96-well U-bottom plate. The cells were incubated with Zombie Aqua dye in the dark for 10 min, then 100 μL of pre-chilled PBS was added to wash. The mixture was centrifuged at 400 g for 5 min, the supernatant was removed, and the cell pellet was collected. Then, 200 μL of fixative was added to the cells, which were then incubated for 20 min to fix. The mixture was centrifuged at 400 g for 5 min, the supernatant was removed, and the cell pellet was gathered. The permeabilization solution was prepared at 1x concentration (diluted with PBS), 200 μL was added to the cell pellet, and the mixture was immediately centrifuged at 400 g for 5 min. The supernatant was discarded and the cell pellet was collected. This process was repeated twice. The prepared antibody mixtures were added to the cells and incubated in the dark for 20 min; then, the cells were washed by adding more 1x permeabilization solution and centrifuging. Finally, the cells were resuspended in fresh PBS (300-500 μL) and transferred to a flow cytometry tube. Flow cytometric analysis was performed using a BD LSRFortessa.

### Method of loading PTX

According to the previously established method for drug loading onto CDEVs (4 mL of CDEVs at a concentration of 1 × 10^10^ particles/mL was combined with 100 μg of PTX, PTX was incorporated into DMSO, sonicated until completely dissolved, and then dispensed to a concentration of 10 mg/mL), the CDEVs were separately mixed with either FITC-PTX or PTX and co-incubated at 37 °C for 1 h^[25]^. Subsequently, drug loading was performed, followed by centrifugal purification. As a result, CDEVs-PTX and CDEVs-FITC-PTX were produced.

The loading efficiency of CDEVs-FITC-PTX was determined using a microplate reader. The supernatant (containing free FITC-PTX) obtained post-centrifugation was used to measure the amount of free FITC-PTX at an absorbance of 495 nm. The precipitate was resuspended to yield CDEVs-FITC-PTX. The loading efficiency of CDEVs-FITC-PTX was calculated using the following formula:

Loading Efficiency = [(Total PTX - free FITC-PTX)/Total FITC-PTX] × 100%

The loading efficiency of CDEVs-PTX was determined using high-performance liquid chromatography (HPLC). One milliliter of CDEVs-PTX (1 × 10^12^) was evaporated at 100 °C. An equal volume of methanol was subsequently added, and the mixture was subjected to vortex and ultrasonic treatment before being sent to the INSTRUMENTAL ANALYSIS CENTER of Shanghai Jiao Tong University for HPLC analysis. All analyses were performed on a C18 column (Supelco Nucleosil C18, 250 mm × 4.6 mm, 5 μm, 100 Å, Sigma-Aldrich) with a mobile phase of H_2_O: acetonitrile (45:55, v/v) at a flow rate of 1 mL/min and a column temperature of 30 °C. Absorbance was measured at 227 nm to monitor the elution of PTX.

### Dye labeling methods

Two different dyes, DiL and DiR, were employed in the experiment. A volume of 1 μL from each dye’s stock solution, which had a concentration of 5 μm, was added to the CDEVs with a particle concentration of 1 × 10^10^ particles/mL. The mixture was then incubated at 37 °C for 1 h. Following incubation, the unbound dyes were removed by centrifugation at 120,000 g for 30 min. The resulting pellets were collected and resuspended in PBS.

### Fluorescence imaging *in vivo*

A volume of 100 μL of Free FITC-PTX and CDEV-FITC-PTX, each with a concentration of 1 × 10^12^ particles/mL, was intraperitoneally injected into mice. At different time intervals after injection (24 and 48 h), the distribution of the FITC-PTX signal within the mice was evaluated through *in vivo* imaging using a Spectral Live Imaging System (IVIS Lumina III). Following these imaging sessions, the mice were sacrificed, and their organs were collected. The IVIS system was then utilized to determine the intensity of the FITC-PTX signal in the excised organs.

### Treatment of tumors *in vivo*

Subcutaneous injections of the Lewis lung carcinoma (LLC) cell line (8 × 10^5^ cells per mouse) were administered to C57BL/6 black mice. After 10 days, four groups of mice (*n* = 10) were administered the following: PBS, CDEVs, PTX, and CDEVs-PTX. Here, CDEVs-PTX was calculated to have a loading efficiency of 62.5% based on experiments. In a subsequent experiment, 3 mg of PTX was added to 1.3 × 10^12^ CDEVs, and after recovery and resuspension with 1 mL of PBS, CDEVs-PTX was obtained at a concentration of 2 mg/mL of PTX in the solution. Simultaneously, free PTX was administered to the mice at the same concentration of 2 mg/mL. The PTX and CDEVs-PTX groups of mice were both administered PTX at a dosage of 100 ug/kg. The intraperitoneal drug administration cycle consisted of five days of administration followed by three days of rest. After three cycles, five mice from each group were sacrificed, and their tumors were collected to measure weight and volume. Portions of the tumor samples were prepared for Hematoxylin and Eosin (HE) staining as well as immunohistochemical analysis. Blood, spleen, and another part of the tumor were separately processed into single cells, incubated with antibodies, and then analyzed using flow cytometry.

The remaining mice (*n* = 5) continued the same treatment regimen. The survival rate was recorded daily, and the experiment was terminated after a total of 50 days of treatment. The surviving mice were then euthanized.

### Cell lines

A549, HCC15, H520, LLC, and Jurkat cells were maintained in cell and tissue culture dishes (Tissue culture treated) within a cell incubator set to 37 °C and 5% CO_2_, with Jurkat cells being grown in suspension. For culturing, A549 and LLC cells were placed in DMEM, while HCC15, H520, and Jurkat cells were cultured in RPMI 1,640 medium. Each type of medium was supplemented with 100 U/mL penicillin, 100 U/mL streptomycin, and 10% heat-inactivated fetal bovine serum, and subsequently filtered through a 0.22 μm filter membrane.

### Mice

C57BL/6 mice, aged 5-6 weeks, were housed in a pathogen-free environment and provided with *ad libitum* feeding. The animal care and use procedures received approval from the Ethics Committee of the School of Biomedical Engineering at Shanghai Jiao Tong University, and all relevant institutional and governmental regulations regarding the ethical use of animals were adhered to.

### Data analysis

The data in this study were evaluated using one-way analysis of variance (ANOVA) or unpaired *t*-tests to identify group differences, with the analyses performed using GraphPad Prism 8.0. Statistical significance was defined as a *P*-value less than 0.05. All results in this paper are expressed as means ± standard deviations, based on a minimum of three independent experiments.

## RESULTS

### CDEVs isolation and their immune modulation

**Figure 1:**
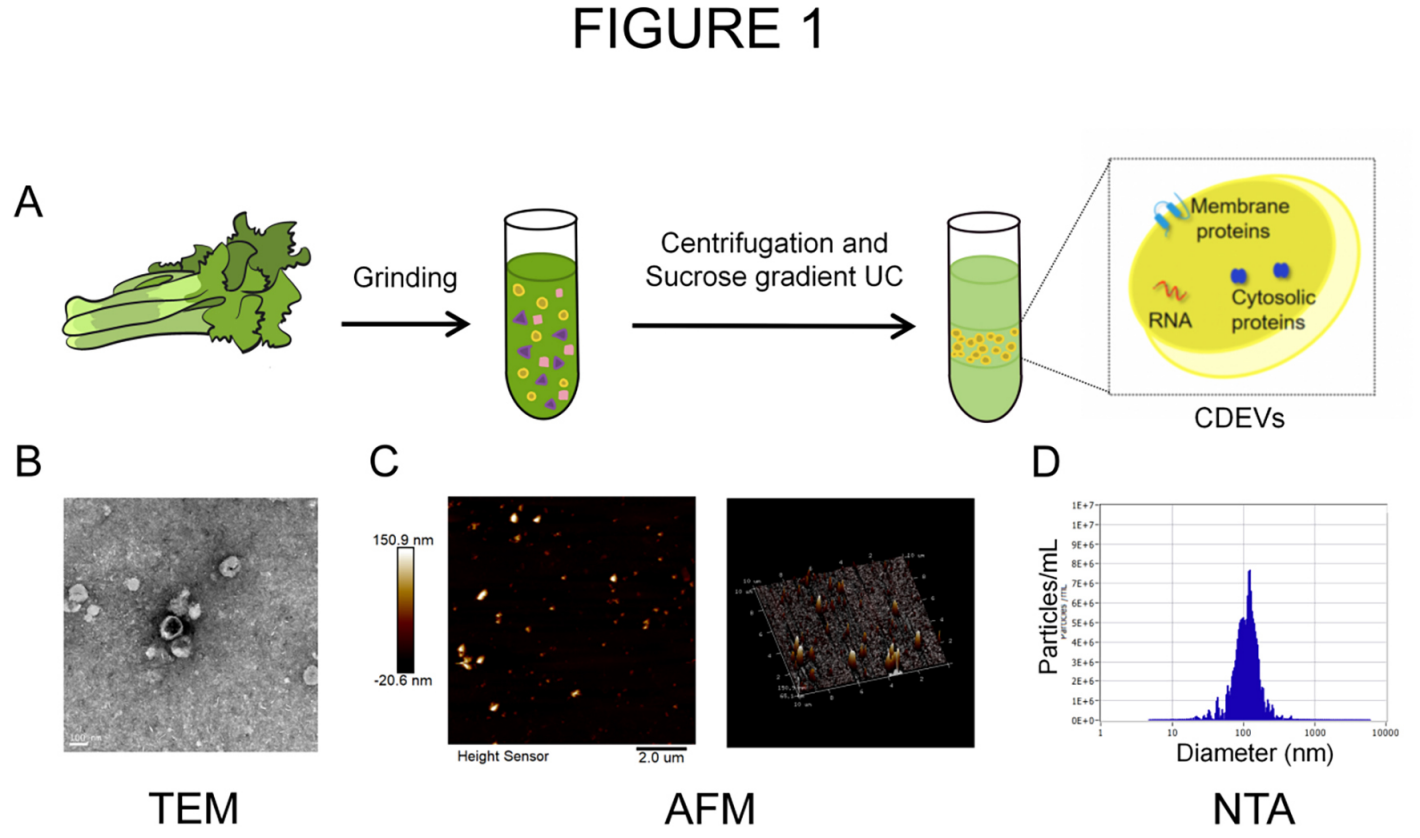
Extraction and characterization of CDEVs. (A) CDEVs are obtained through the centrifugation methods; (B-D) The characterization results of CDEVs using TEM, NTA, and AFM. Scale bar of (B): 100 nm; Scale bar of (C): 2.0 μm. CDEVs: Celery-derived extracellular vesicles; TEM: transmission electron microscopy; NTA: Nanoparticle Tracking Analysis; AFM: atomic force microscopy; UC: ultracentrifugation.

Our previous research found that PDEVs can be taken up by tumor cells^[25]^. Among the extracellular vesicles extracted from lemon, grape, ginger, and celery, those extracted from celery exhibited the highest cellular uptake rate [Supplementary Figure 1A]. Upon juicing the celery using a wall-breaker, CDEVs were subsequently extracted via a series of procedures including centrifugation and ultracentrifugation [Figure 1A]. Subsequently, the extracted CDEVs were characterized using techniques such as TEM, NTA, and AFM. TEM revealed that the CDEVs were spherical entities with a double-membrane structure, exhibiting a diameter of approximately 100 nm [Figure 1B]. Figure 1C presents 2D and 3D images obtained via AFM scanning, illustrating that the CDEVs exhibit no aggregation or deformation. Additionally, the 3D image provided height information of CDEVs. NTA demonstrated that the diameter of the CDEVs ranges between 100-200 nm [Figure 1D].

**Figure 2:**
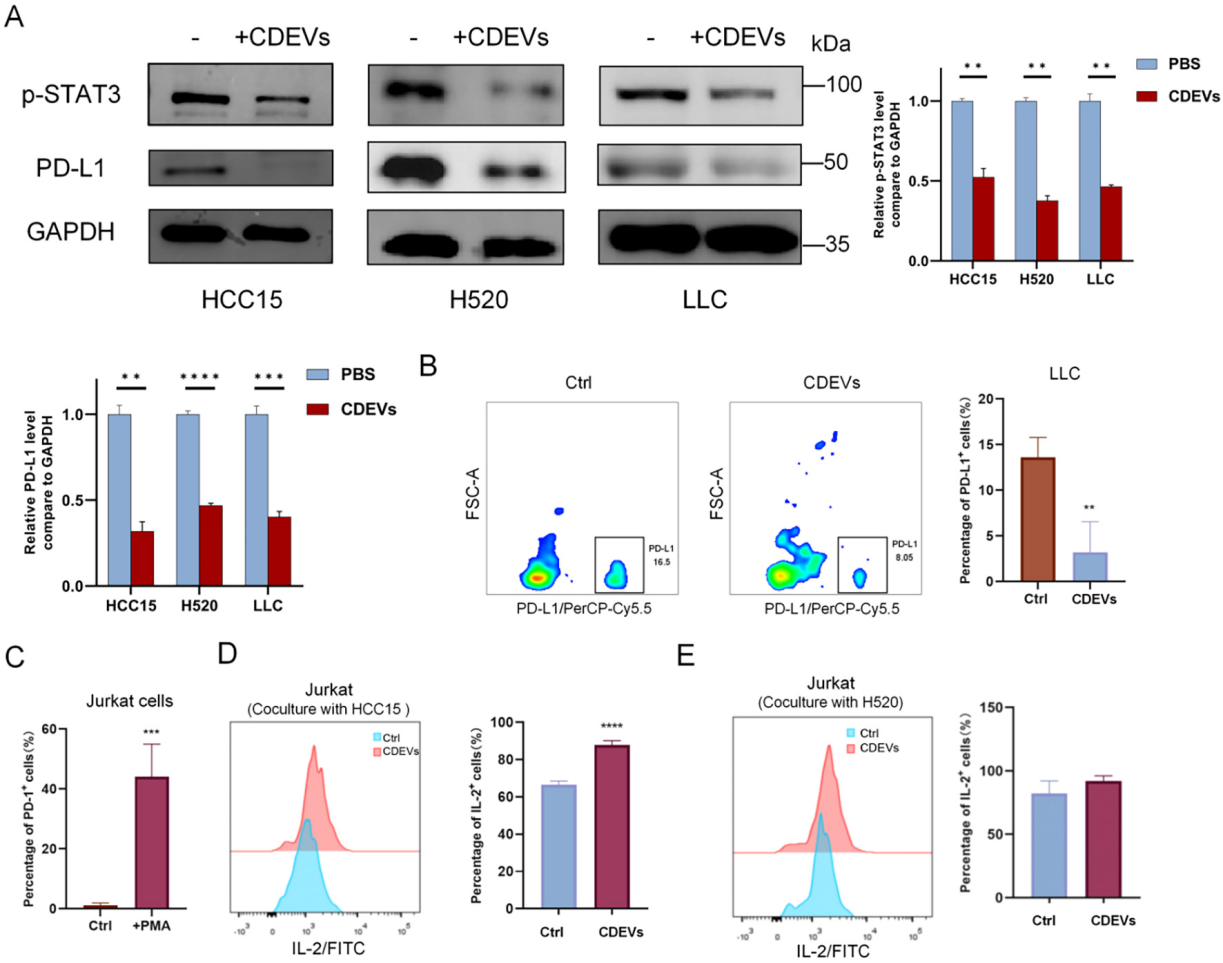
CDEVs have the function of inhibiting the expression of PD-L1 in lung cancer cell lines. (A) Western blot results show that the addition of CDEVs can inhibit the expression of PD-L1 and p-STAT3 in HCC15, H520, and LLC cell lines; (B) Flow cytometry results show a decrease in PD-L1 expression in LLC cells after the addition of CDEVs; (C) Flow cytometry results demonstrate a significant increase in PD-1 expression in Jurkat cells after PMA stimulation; (D and E) Secreted IL-2 from Jurkat-mediated HCC15 or H520 assays was measured using flow cytometry. Data are represented as mean ± SD. LLC: Lewis lung carcinoma; CDEVs: celery-derived extracellular vesicles; HCC15: human pulmonary adenocarcinoma cell line; H520: human lung squamous cell carcinoma cell line; PD-L1: programmed cell death-Ligand 1; p-STAT3: phosphorylated signal transducer and activator of transcription 3; PMA: Phorbol 12-Myristate 13-Acetate. (*n* = 4; ^*^P < 0.05; ^**^P < 0.01; ^***^P < 0.01; ^***^P < 0.001).

To investigate the effects of CDEVs on different lung cancer cell lines, we added CDEVs to HCC15, H520, LLC, and A549 cells, respectively, and treated them for 48 h. According to the western blot results [Figure 2A] and flow cytometry data [Figure 2B], the expression of PD-L1 on the surface of the cells to which CDEVs were added was decreased, and this decrease was dose- and time-dependent [Supplementary Figure 1B-E]. Meanwhile, p-STAT3 expression also produced a decrease with the addition of CDEVs [Figure 2A].

PD-L1 is a ligand for the immunosuppressive receptor PD-1. Lung cancer cells can inhibit T cell activity through PD-L1/PD-1 interaction. Therefore, we subsequently explored the effect of CDEVs on the interaction between Jurkat T cells (expressing PD-1) and lung cancer cells (expressing PD-L1) *in vitro*. The data [Figure 2C and Supplementary Figure 1F] show a high expression of PD-1 after the addition of the stimulant PMA in Jurkat T cells. PD-L1 on cancer cells affected the activation of Jurkat T cells by binding to the PD-1 receptor on activated T cells, resulting in decreased IL-2 expression^[27,28]^. IL-2 expression rose in the group with CDEVs added [Figure 2D and E], implying that Jurkat T cell activity, which should have been inhibited by PD-L1, was activated.

### The biodistribution of different drugs *in vivo*

The distribution of DiR-labeled CDEVs in the tissues of mice was consistent between the intraperitoneal and intravenous administration groups^[25]^, so we subsequently investigated the distribution of DiR-labeled CDEVs in mice under tail-vein injection conditions only. The concentration of CDEVs in the blood decreased substantially 5 min after injection [Supplementary Figure 2A and B], but the concentration of CDEVs that could be monitored in the subsequent time did not change much because of the limit of detection of IVIS. Supplementary Figure 2C-E also show the results of *in vivo* versus *ex vivo* organ distribution. Notably, signals from CDEVs were detected in the mouse brain within 24 h after injection [Supplementary Figure 2F]. This suggests that CDEVs can cross the blood-brain barrier.

**Figure 3:**
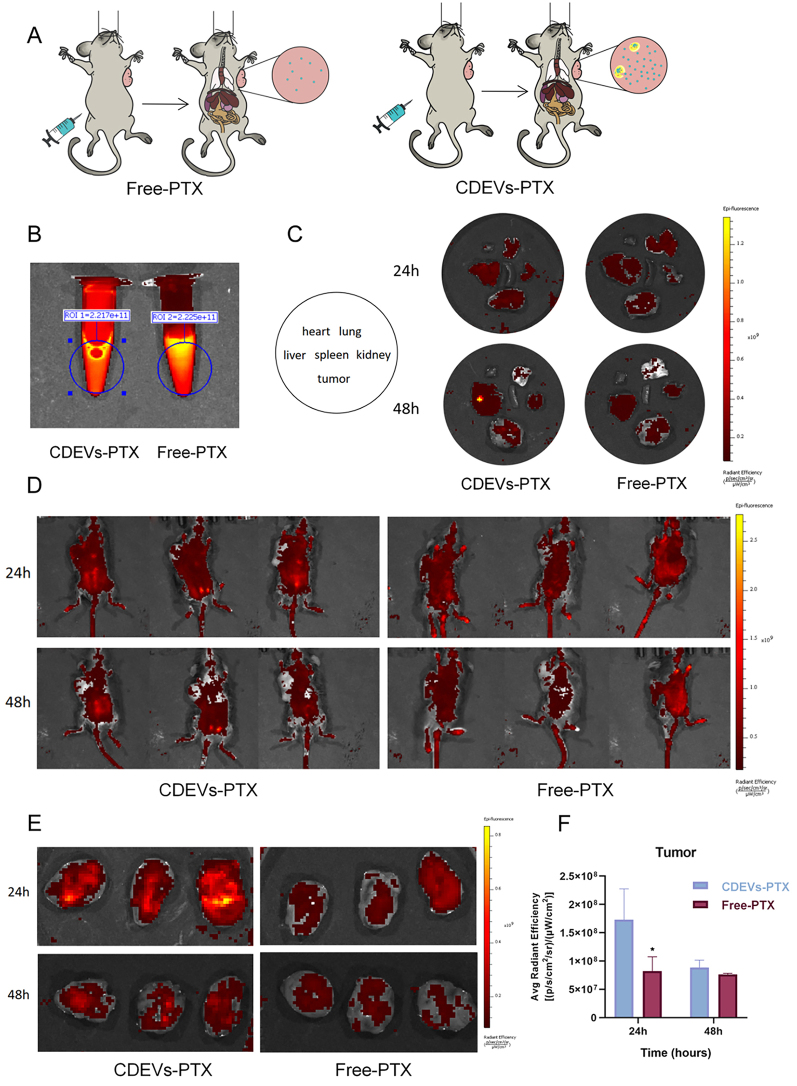
The *in vivo* distribution of free PTX and PTX encapsulated in CDEVs. (A) Schematic representation of *in vivo* distribution experiment in LLC tumor-bearing mice; (B) IVIS imaging of CDEVs-PTX and PTX; (C) The distribution of CDEVs-PTX and PTX in organs and tumors at 24 and 48 h post-administration; (D)*In vivo* distribution of CDEVs-PTX (A) and PTX (B) at 24 and 48 hours post-administration; (E and F) Tumor distribution and fluorescence signal quantification results of CDEVs-PTX and PTX. Data are represented as mean ± SD. CDEVs: Celery-derived extracellular vesicles; PTX: paclitaxel; LLC: Lewis lung carcinoma. (*n* = 3; ^*^P < 0.05).

To investigate the tissue tropism of CDEVs loaded with FITC-PTX and free FITC-PTX, we assessed the *in vivo* biodistribution of these two PTX groups in tumor-bearing mice using a small animal imaging system [Figure 3A]. In these studies, we first evaluated the FITC fluorescence intensity of PTX after wrapping by CDEVs versus free PTX, and the results showed no change in FITC fluorescence intensity after being wrapped by CDEVs [Figure 3B]. At 24 and 48 h following intraperitoneal injection, FITC signals in both groups were detected mainly in the liver, lung, kidney, and tumor tissues [Figure 3C]. Regarding *in vivo* distribution [Figure 3D], the signals of the intraperitoneal injection group were diffusely distributed in the abdominal cavity and the distribution of the signals in the two groups was relatively consistent. However, Figure 3E and F showed that the CDEVs-FITC-PTX signals were stronger in the mouse tumors 24 h after injection compared to the free FITC-PTX group. This suggests that PTX encapsulated by CDEVs can be enriched more in the tumor site, owing to the enhanced permeability and retention (EPR) effect.

### The use of CDEVs-PTX demonstrated a better antitumor treatment effect

**Figure 4:**
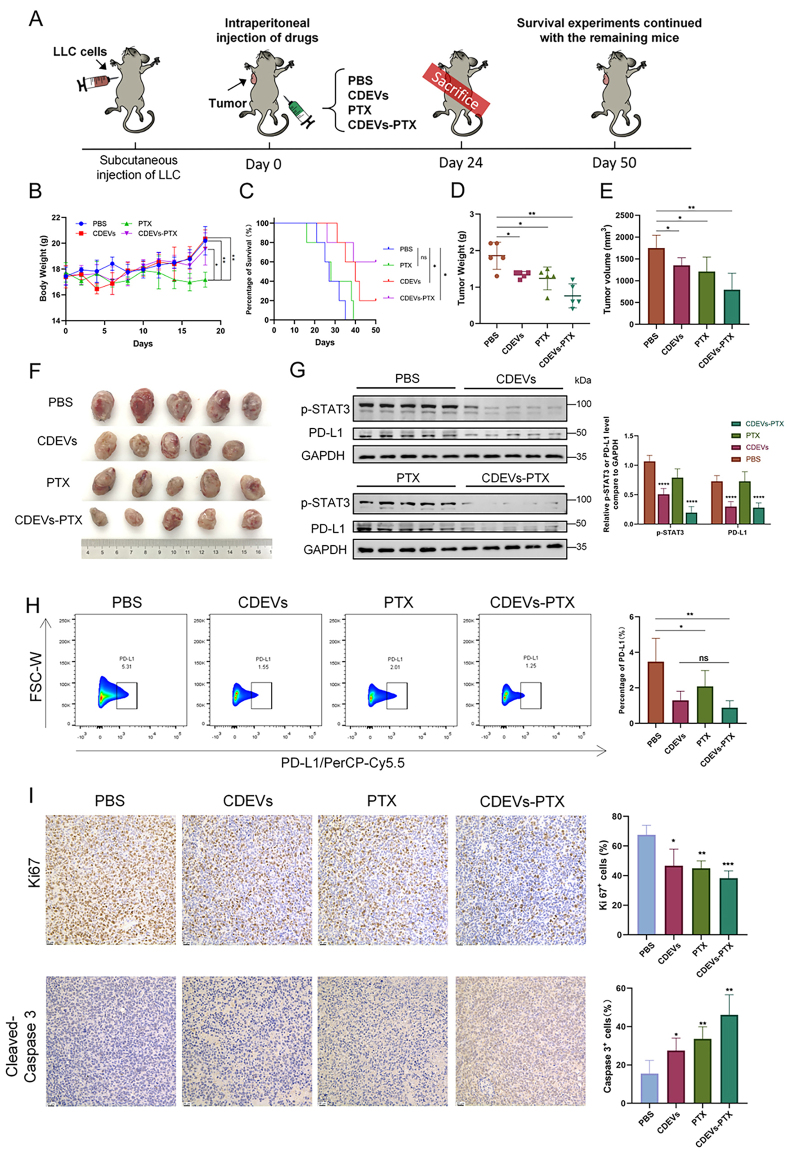
CDEVs, PTX and CDEVs-PTX for tumor therapy *in vivo*. Their antitumor effects were assessed across four groups (*n* = 5). (A) Schematic representation of *in vivo* tumor therapy experiment in mice; (B and C) Body weight and survival rate in treated mice; (D and E) The size and weight of tumors in each group were assessed; (F) Tumor images were captured for each group; (G) Western blot data about the expression of PD-L1 in each group; (H) Flow cytometry data on PD-L1 expression were analyzed for each group; (I) Immunohistochemical tumor sections from the four groups were stained with Ki-67 and Cleaved-Caspase 3 markers. The sections show the positive staining regions for Ki-67 (upper panel) and Cleaved-Caspase 3 (lower panel). Scale bar: 100 μm. Data are represented as mean ± SD. CDEVs: Celery-derived extracellular vesicles; PTX: paclitaxel; CDEVs-PTX: CDEVs loaded with PTX; PBS: phosphate buffer solution. (*n* = 4; ^*^P < 0.05; ^**^P < 0.01; ^***^P < 0.01; ^***^P < 0.001).

We explored the potential of using CDEVs in combination with PTX for tumor treatment. In C57BL/6 mice with normal immunity, we compared the therapeutic effects of four different treatment groups: PBS, CDEVs, PTX, and CDEVs-PTX. The subcutaneous tumor implantation and treatment plans are depicted in Figure 4A. TEM revealed that the morphology of CDEVs-PTX retained their structure, with a diameter remaining around 100 nm [Supplementary Figure 3A].

Figure 4B reveals that compared to the other three groups, the body weight of mice in the PTX group significantly decreased, implying toxicity of PTX to mice. The toxicity of PTX encapsulated by CDEVs was significantly reduced, which also contributed to the improved survival rate of the tumor-bearing mice [Figure 4C]. It is worth mentioning that the CDEVs group, compared to the PBS group, could also inhibit tumor growth [Figure 4D-F] and prolong survival, possibly due to the reduction in the expression of PD-L1 in LLC cells by CDEVs. Western Blot results [Figure 4G] and flow cytometry data [Figure 4H] showed that the expression of PD-L1 in the tumors of mice in both the CDEVs group and the CDEVs-PTX group decreased, and these two groups showed significant differences in expression levels compared to the PBS group [Figure 4H]. Subsequently, the antitumor effects of CDEVs, CDEVs-PTX, and free PTX in the C57BL/6 mouse subcutaneous tumor model were examined by staining tumor tissues with the cell proliferation marker Ki67 and the apoptosis marker Cleaved-Caspase 3. At the same time, we also conducted HE staining of the samples [Supplementary Figure 3B]. The results of IHC [Figure 4I] showed that the Ki67-positive area in the tumors treated with PBS was much higher than in the other three groups, with the lowest positivity rate in the CDEVs-PTX group. As for Cleaved-Caspase 3, the CDEVs-PTX group had the highest positive area. Compared to the PBS group, CDEVs, PTX, and CDEVs-PTX all exhibited inhibitory effects on tumor growth. Among them, CDEVs-PTX presented the best therapeutic effect, which means that the combination of CDEVs and PTX can improve the therapeutic effect of either entity alone.

### CDEVs can reduce PD-L1 expression levels and activate CD8+ T cells

**Figure 5:**
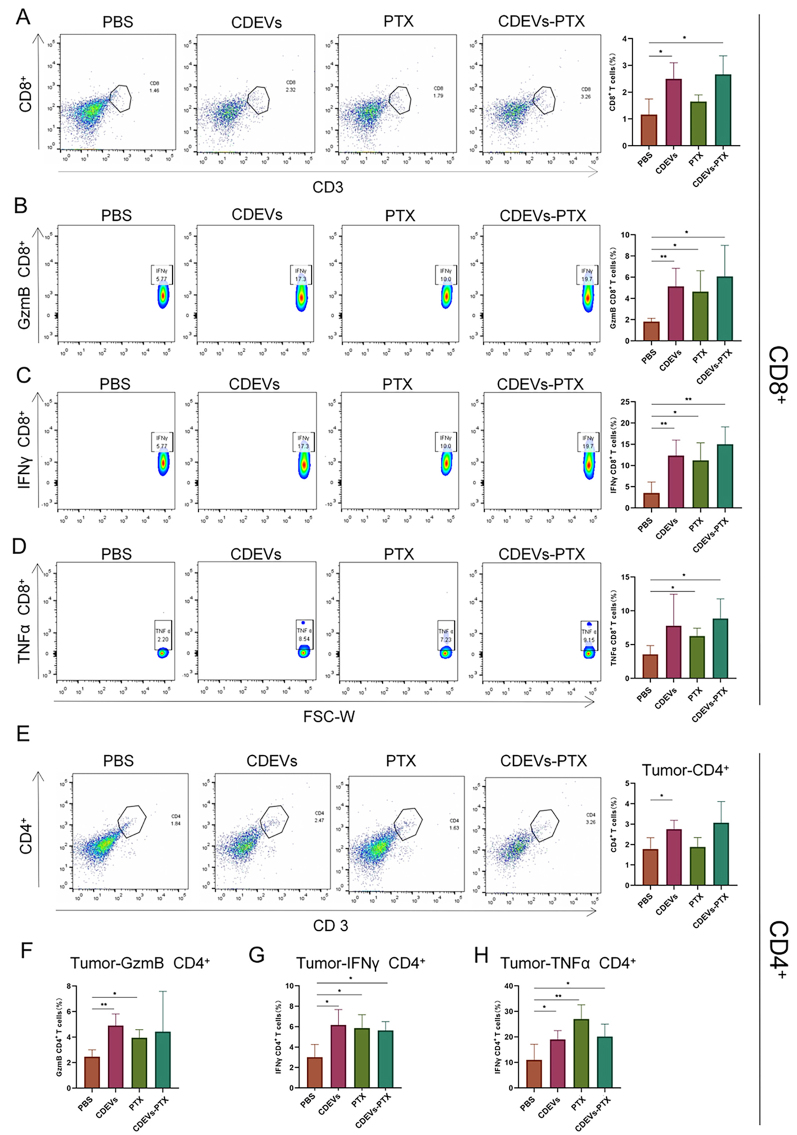
CDEVs function as an immunotherapy in tumors. (A) Data for the proportion of tumor-infiltrating CD8+ T cells; (B-D) Data for the proportion of GzmB, IFN-γ, and TNF-α secreted by tumor-infiltrating CD8+ T cells; (E) Data for the proportion of tumor-infiltrating CD4+ T cells; (F-H) Data for the proportion of GzmB, IFN-γ, and TNF-α secreted by tumor-infiltrating CD4+ T cells. Data are represented as mean ± SD. CDEVs: Celery-derived extracellular vesicles; PTX: paclitaxel; CDEVs-PTX: CDEVs loaded with PTX; PBS: phosphate buffer solution. (*n* = 4; ^*^P < 0.05; ^**^P < 0.01; ^***^P < 0.01; ^***^P < 0.001).

After the above treatments, lymphocytes were collected from the spleen and tumor tissues of mice for flow cytometry analysis. Subsequently, tumor-infiltrating T cells were analyzed in the four treatment groups. Activated CD8+ T cells in the tumor microenvironment have anticancer immunity, So we first assessed changes in the number of CD8+ T cells infiltrated by the tumor. The groups of CDEVs and CDEVs-PTX showed increased cell numbers compared with the PBS and PTX groups [Figure 5A]. Typical CD8+ T cells exhibiting cytotoxic activity are characterized by their high expression of granzyme B (GzmB), IFN-γ and TNF-α. The groups of CDEVs and CDEVs-PTX showed a rise in the production of GzmB, IFN-γ, and TNF-α, which provides support for the good tumor suppression effect [Figure 5B-D]. The percentage of CD4+ T cells was higher in the CDEVs and CDEVs-PTX groups than in the PBS group [Figure 5E]. The amounts of the three cytokines in CD4+ T cells rose in the CDEVs and CDEVs-PTX groups [Figure 5F-H].

Simultaneously, an increase in the CD8+ T cell population was observed in the spleen of mice from both the CDEVs and CDEVs-PTX groups [Supplementary Figure 4A], along with elevated secretion levels of GzmB, IFN-γ, and TNF-α [Supplementary Figures 4B]. And there was an increase in CD4+ T cells in the spleen [Supplementary Figure 4C]. The variation in CD4+ T cells suggests that both helper and cytotoxic functions of CD4+ T cells contribute to tumor suppression.

## DISCUSSION

Compared to animal cell-derived extracellular vesicles, PDEVs have certain advantages, such as low production costs, low immunogenicity, and stability, and are known to be taken up by numerous cell types^[26,29]^. Furthermore, PDEVs possess inherent therapeutic effects due to their natural active ingredients. However, PDEVs still have areas that require further exploration and research^[30]^. Firstly, the standardization of PDEVs’ production methods needs to be established. Secondly, the interaction mechanism between PDEVs and recipient cells remains unclear. It is yet to be determined whether the communication between PDEVs and recipient cells is specific or random, and ambiguity still exists in this aspect. Additionally, PDEVs may exhibit reduced tissue targeting ability compared to extracellular vesicles since they do not originate from animals. Therefore, further research is required to enhance our understanding of the biological activity, applications, and generation of PDEVs.

CDEVs, when serving as drug carriers loaded with chemotherapeutic drugs, enhance therapeutic efficacy while reducing toxic side effects^[25]^. This improvement is particularly important because chemotherapeutic drugs are less stable, prone to decomposition or inactivation *in vivo*, and lack targeting properties, which can lead to widespread distribution in the body, aggravating toxic side effects^[14]^. In our experiments, the better tumor-targeting effect of PTX loaded by CDEVs can be attributed to the following two factors. First, the lipid bilayer structure of CDEVs provides PTX with enhanced stability *in vivo* and improved sustained release effects^[26]^. Second, the targeting mechanisms of nanoparticles can be divided into passive and active targeting^[31]^. Passive targeting refers to the accumulation of small-sized nanoparticles (50-200 nm) at tumor sites through leakage from the surrounding tumor blood vessels, known as the EPR effect^[32,33]^. This mechanism leverages the circulation characteristics of nanoparticles in the bloodstream, making their accumulation in tumor tissues more effective^[34]^. Studies have shown that PDEVs can also accumulate more in tumor sites by enhancing the EPR effect^[20]^. Furthermore, the EPR effect has demonstrated therapeutic advantages in the clinical application of nanoparticles carrying chemotherapeutic drugs^[25,35]^. So, the nano-size of CDEVs allows them to enter tumor tissues through these abnormally leaky vessel walls, which makes them more likely to accumulate at the tumor site, thus reducing the effect of PTX on healthy tissues.

CDEVs themselves, as a therapeutic agent, impact the immune microenvironment by downregulating PD-L1 expression. This enhances the cytotoxic activity of tumor-infiltrating CD8+ T cells and promotes the secretion of GzmB, IFN-γ, and TNF-α. In coordination with CD4+ T cells, this suppresses tumor progression^[36]^. It is worth noting that the decrease in PD-L1 is accompanied by a reduction in the p-STAT3 signal as well^[7]^. STAT3 is significantly activated in lung cancer and is essential in driving tumor advancement^[37]^. The activation of STAT3 is also essential for the growth, metastasis, and angiogenesis of many tumors^[38]^. However, the incomplete data on plant protein libraries and the insufficient exploration of molecular mechanisms prevent us from fully elucidating the precise mechanisms of CDEVs’ action. Moreover, the specific active molecules involved have yet to be identified. In the future, we aim to explore in-depth the mechanisms by which CDEVs influence p-STAT3 and other related pathways.

The research demonstrates that CDEVs can serve not only as carriers for drug delivery, effectively loading chemotherapy drugs, but also correlate with downregulated PD-L1 expression, which may enhance the killing function of immune-infiltrating CD8+ T cells in lung cancer^[36,39]^. By combining these two functionalities, CDEVs can achieve the combined therapeutic effects of immunotherapy and chemotherapy in a single administration.

## DECLARATIONS

### Availability of data and materials

The datasets used and analyzed during the current study are available from the corresponding author upon reasonable request.

### Copyright

© The Author(s) 2024.
