# Supplementary material for: The unexpected PD-L1 suppression function of celery-derived extracellular vesicles improves lung cancer chemotherapy efficacy
Source: Extracell Vesicles Circ Nucl Acids. 2024 Nov 9;5(4):597–613. doi: 10.20517/evcna.2023.75 (PMC11725424; doi:10.20517/evcna.2023.75)
Supplement: Supplementary file 1 [file evcna-5-4-597-SupplementaryMaterials.pdf]

Supplementary Materials

The unexpected PD-L1 suppression function of celery-derived extracellular vesicles improves lung cancer chemotherapy efficacy

Xin Lu#, Ziwen Yu#, Junjun Wang, Ao Tian, Tingyu Wu, Yirui Cheng, Qing Han, Fan Li, Weiliang Xia

State Key Laboratory of Systems Medicine for Cancer, Renji Hospital, School of Medicine and School of Biomedical Engineering, Shanghai Jiao Tong University, Shanghai 200030, China.

#Authors contributed equally.

Correspondence to: Prof. Weiliang Xia, State Key Laboratory of Systems Medicine for Cancer, Renji Hospital, School of Medicine and School of Biomedical Engineering, Shanghai Jiao Tong University, 1954 Huashan Road, Shanghai 200030, China. E-mail: wlxia@sjtu.edu.cn

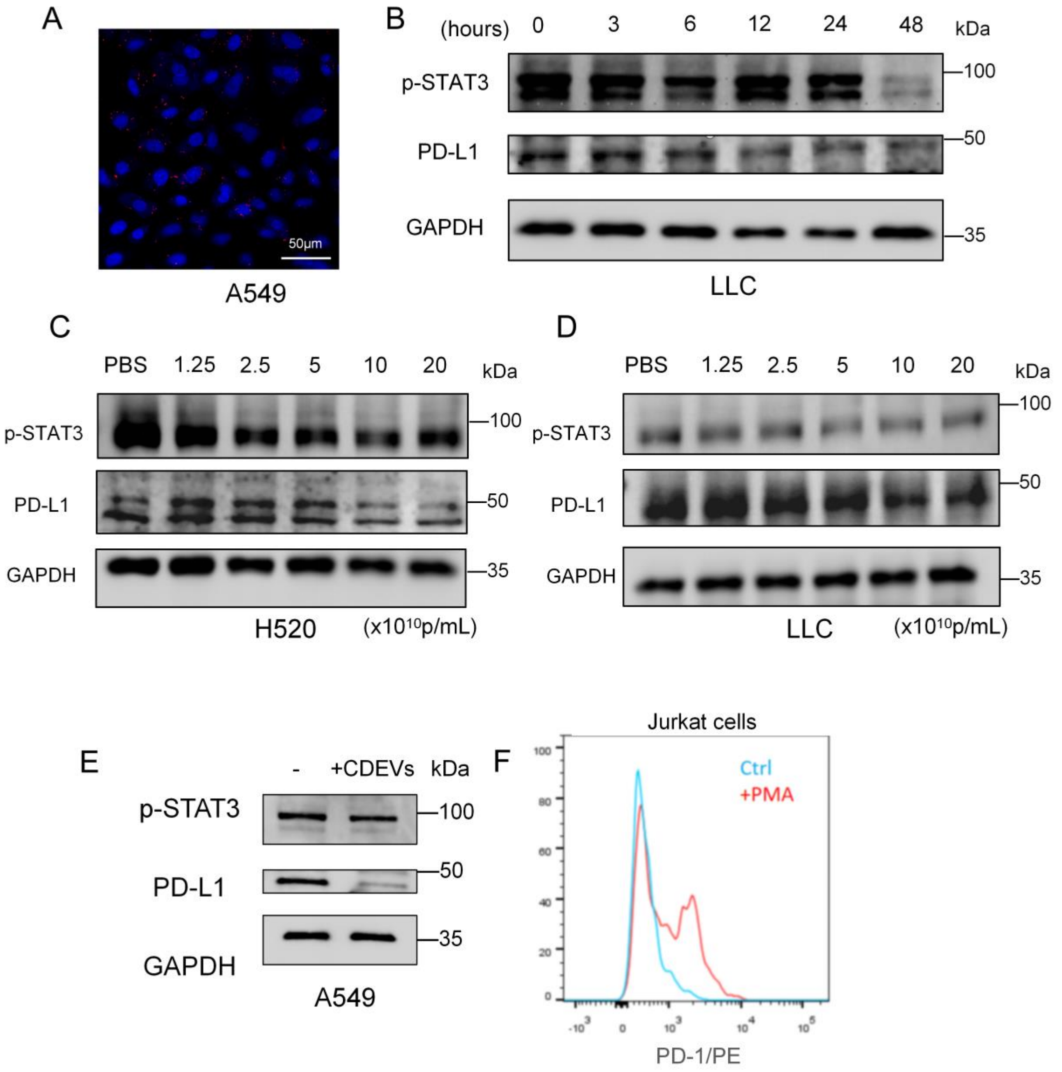

Supplementary Figure 1. (A) CDEVs (Dil, Red) can be taken up by cells (DAPI, Blue); (B-D) WB of cells treated with different concentrations (C-D) and different time points (B) of adding CDEVs; (E) WB results show that the addition of CDEVs can inhibit the expression of PD-L1 and p-STAT3 in A549 cell lines. F. flow cytometry result of Figure 2C.

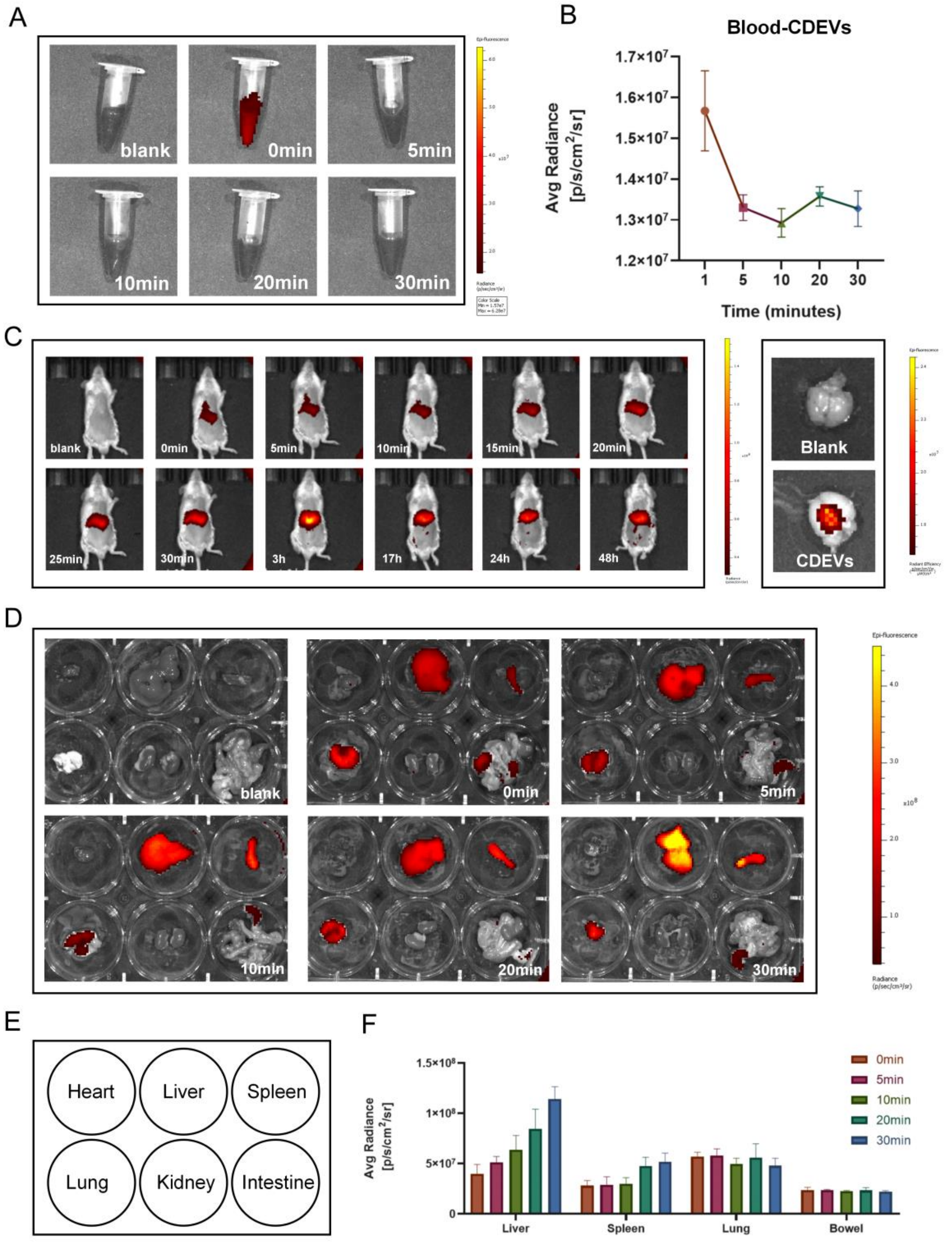

Supplementary Figure 2. (A-B) Changes of DIR-labeled CDEVs signal levels in mouse blood (A) with their quantitative results (B); (C) DIR-labeled CDEVs signal in mice at shorter time points and in mouse brain 30 min after administration; (D-F) DIR-labeled CDEVs signals in mouse organs (D-E) with their quantitative results (F).

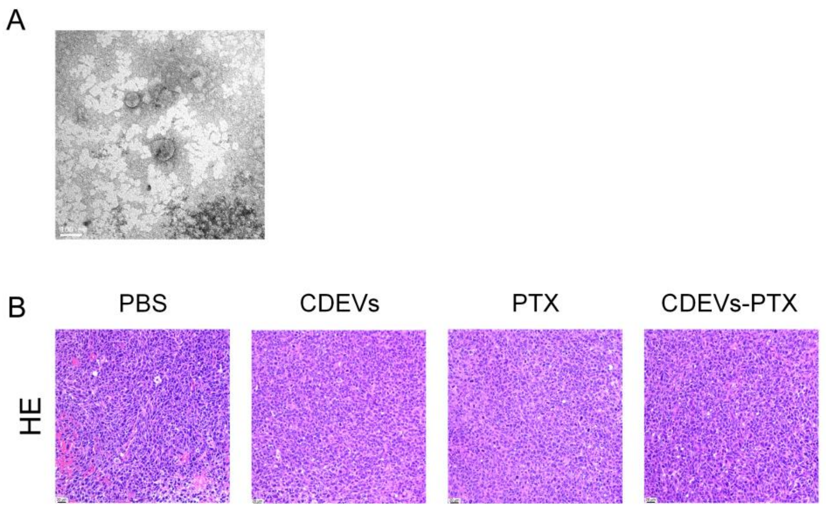

Supplementary Figure 3. (A) TEM figure of CDEVs-PTX; (B) HE staining of tumors were obtained from the four groups.

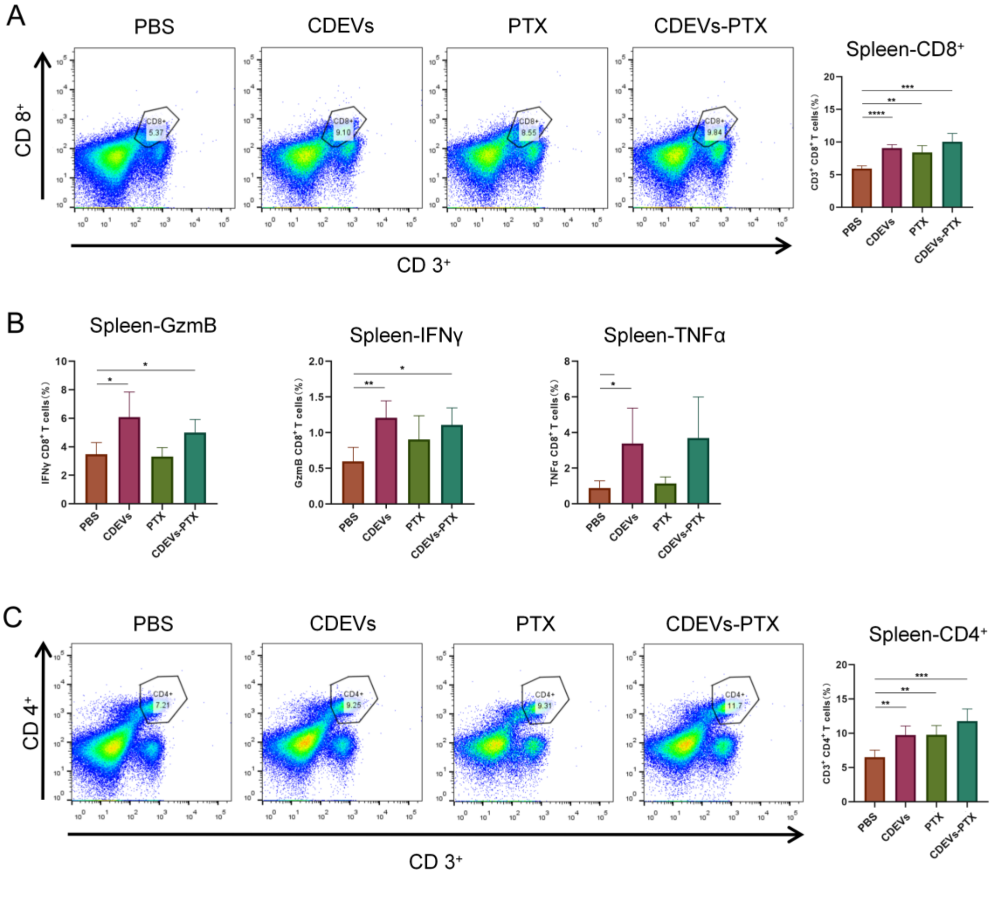

Supplementary Figure 4. (A) Flow cytometry analysis for the proportion of CD8+ T cells in the spleen; (B) Flow cytometry analyses of the proportion of GzmB-, IFNγ- and TNFα-producing CD8+ T cells in the spleen; C: Flow cytometry analysis for the proportion of CD4+ T cells in the spleen.
